# Nanoantenna induced liquid crystal alignment for high performance tunable metasurface

**DOI:** 10.1515/nanoph-2023-0446

**Published:** 2023-09-26

**Authors:** Rasna Maruthiyodan Veetil, Xuewu Xu, Jayasri Dontabhaktuni, Xinan Liang, Arseniy I. Kuznetsov, Ramon Paniagua-Dominguez

**Affiliations:** Institute of Materials Research and Engineering (IMRE), Agency for Science, Technology and Research (A*STAR), 2 Fusionopolis Way, Innovis #08-03, Singapore 138634, Republic of Singapore; Department of Physics, Ecole Centrale School of Engineering, Mahindra University, Hyderabad 500 043, India

**Keywords:** liquid crystals, spatial light modulators, nanoantenna, metasurface, resonance, response time

## Abstract

Liquid crystal (LC) based spatial light modulators (SLMs) are a type of versatile device capable of arbitrarily reconfiguring the wavefront of light. For current commercial LC-SLM devices, the large pixel size limits their application to diffractive optics and 3D holographic displays. Pixel miniaturization of these devices is challenging due to emerging inter-pixel crosstalk, ultimately linked to the thick LC layer necessary for full phase (or amplitude) control. Integration of metasurfaces, i.e., 2D arrangements of resonant nanoantennas, with thin LC has emerged as a promising platform to boost light modulation, enabling realization of sub-wavelength pixel size SLMs with full phase (or amplitude) control. In most devices realized so far, however, the presence of an alignment layer, necessary to induce a preferential initial LC orientation, increases the voltage requirement for resonance tuning and reduces the efficiency of light modulation, something that accentuates for an ultra-thin (e.g., submicron) metasurface-LC cell. Here, we present an alternative strategy by which the LC molecular alignment is purely controlled by the periodicity and geometry of the nanoantenna without any additional alignment layer. The nanoantennas are specifically designed for the double purpose of sustaining optical resonances that are used for light modulation and to, simultaneously, induce the required LC pre-alignment. The proposed device structure allows lower voltage and reduced switching times (sub-millisecond) compared to devices including the alignment layer. This novel strategy thus helps to improve the performance of these miniaturized-pixel devices, which have emerged as one of the potential candidates for the next generation of products in a wide range of applications, from virtual/augmented reality (VR/AR) and solid-state light detection and ranging (LiDAR), to 3D holographic displays and beyond.

## 1 Introduction

Liquid crystal (LC) based spatial light modulator (SLM) is one of the most prevalent technologies for reconfiguring the wavefront of light. LC-SLMs consist of a one- or two-dimensional array of pixels which are capable of locally and arbitrarily manipulating the wavefront of light. They use the birefringent properties of LC to modulate the amplitude, phase, or polarization of light on top of each pixel via application of electrical biases [1, 2]. They have been widely used for 2D projection displays and optical communication. However, their great potential for applications to large field diffractive optics and 3D holographic displays has not been unleashed due to their large pixel size, which limits the highest achievable diffraction angle and field of view (FOV). The large pixel size also causes high order diffraction, which, most of the time, deteriorates the performance of the device, and extra optics are needed to filter it out. Therefore, LC-SLM with smaller pixel size is preferred for these applications. However, pixel size miniaturization of LC-SLM devices is challenging due to emerging inter-pixel crosstalk, which is the unwanted light modulation in the adjacent pixels to any given one that is purposedly addressed, and which arises due to the spreading of the applied electric field [3–6]. For phase modulating SLMs, the phase delay is accomplished by the orientation of LC molecules at individual pixel level, which thus changes the refractive index experienced by the light along the propagation path and, in turn, controls the phase shift (or retardation). The phase coverage is proportional to the thickness of the LC medium. For transmissive and reflective SLMs the LC thickness should be at least *λ*/Δ *n* and *λ*/2Δ*n*, respectively, to achieve 2*π* phase modulation, where Δ*n* is the birefringence of the LC (defined as the difference between the real part of its extraordinary and ordinary refractive indices) and *λ* the operational wavelength. The response speed of the device is proportional to the square of the LC thickness under the strong anchoring condition and varies linearly with LC thickness for weak anchoring [7, 8]. The inter pixel cross talk sets a limit to the minimum pixel size of the device and is in turn also determined by the LC thickness. The LC thickness is thus a critical parameter in these devices, as it constrains the pixel size and response speed, which subsequently define their FOV and refresh rate. Therefore, reducing the LC thickness turns out to be crucial for some of the versatile applications of SLMs, such as light detection and ranging (LIDAR), augmented/virtual reality (AR/VR), and holographic displays, to name a few, where both FOV and speed are critical.

Metasurfaces, i.e., 2D arrangements of subwavelength elements (also known as nanoantennas) able to locally modify the properties of light, have emerged as a promising platform for nanophotonic applications for wavefront manipulation [9–19]. Different types of materials, including metallic [19–23], dielectric and semiconductor [24–29] or even phase change materials can be used to fabricate the nanoantennas. Among them, all-dielectric and semiconductor metasurfaces are those shown to manipulate electromagnetic wavefronts most efficiently due to their negligible losses. These can be achieved either non-resonantly, via waveguiding or using the geometric phase, or by the excitation of localized (Mie-type) or collective (non-local) resonances [24–28, 30–35]. In the resonant case, the wavefront modulation properties of a metasurface can be changed from static to dynamic using external stimuli, either to modify the material properties of the nanoantennas themselves [36–41] or their immediate environment [42–48]. For the latter case, LC is a promising candidate due to its wide range tunability of permittivity, its transparency at optical frequencies, and the possibility to address it using electric fields, magnetic fields, or by thermal phase transition [30–35, 45–48]. In the arguably most practical case of electrical biasing, the reorientation of LC molecules either parallel or perpendicular to the applied electric field (depending on the addressing scheme) locally modifies the near-field environment of the nanoantennas, enabling highly efficient spectral tuning of their resonances and the associated phase modulation.

In the past few years, it has been shown that all-dielectric metasurfaces integrated with traditional LC device architectures allow pixel size miniaturization [30–35]. This is because, unlike in conventional SLMs, abrupt optical phase shifts in these devices are introduced by the resonant character of the nanoantennas, thus uncoupling the phase modulation from the LC thickness. Ideally, one would like to use localized, Mie-like resonances, minimizing lattice coupling effects, and which can be made to interfere or couple with each other to provide different degrees of freedom for the wavefront modulation [30–35]. LC can then be used for high efficiency spectral tuning of these resonances and the associated phase modulation at the individual pixel level. Since only the near-field of the nanoantennas needs to be modified (which typically extends only a few hundreds of nanometers at optical frequencies), their presence allows to reduce the thickness of the tunable medium (LC) significantly, and thus the crosstalk between the neighboring electrodes, allowing pixel miniaturization [32–35].

Thus, an ideal LC-tunable meta-device would consist of a miniaturized LC cell, with thickness as small as possible as to minimize the crosstalk (in practice, this should imply LC cells of less than 1 μm). For such thin cells, however, several problems arise if one uses the common strategy to induce the pre-alignment of the LC, consisting of using an alignment layer (which is typically either a polyimide or a photoalignment layer). First, for ultra-thin cells, the dielectric shielding effect would be apparent even with a thin alignment layer and the effective phase shift would be reduced. Second, the strong anchoring from the alignment layer for an ultra-thin LC cell significantly slows down the electric field response of the LC and increases the range of voltages required for switching, which might increase again the crosstalk, ultimately hindering the benefit of using thinner cells [49–51]. Thus, for ultra-thin LC cell devices, new LC alignment strategies need to be developed.

Here, we report one such strategy, in which the metasurface (or nanoantennas) are specifically designed for the double purpose of sustaining optical resonances that are used for light modulation and to, simultaneously, induce the required LC pre-alignment. In this scenario, the LC molecular alignment is purely controlled by the periodicity and geometry of the nanoantennas, without any additional alignment layer, thus helping to solve the high driving voltage and the long response time issues mentioned above. This is in line with previous works that showed the impact of the presence of nanoantennas on the alignment of the LC [30, 52, 53] and/or how micro or nanopatterning can be used to induce the LC pre-alignment in some applications [22, 54–60], but done here judiciously to create nanoantennas that serve the double purpose of providing a strong optical resonant response and inducing the required LC orientation. Therefore, the resulting LC-tunable metasurfaces without an alignment layer display lower threshold and saturation voltages, and much faster (sub-millisecond) switching speeds of the LC compared to their counterparts with an alignment layer. As will be shown, such nanoantenna-induced LC alignment is only possible due to the miniaturization of the cell thickness.

## 2 Results and discussions

**Figure 1:**
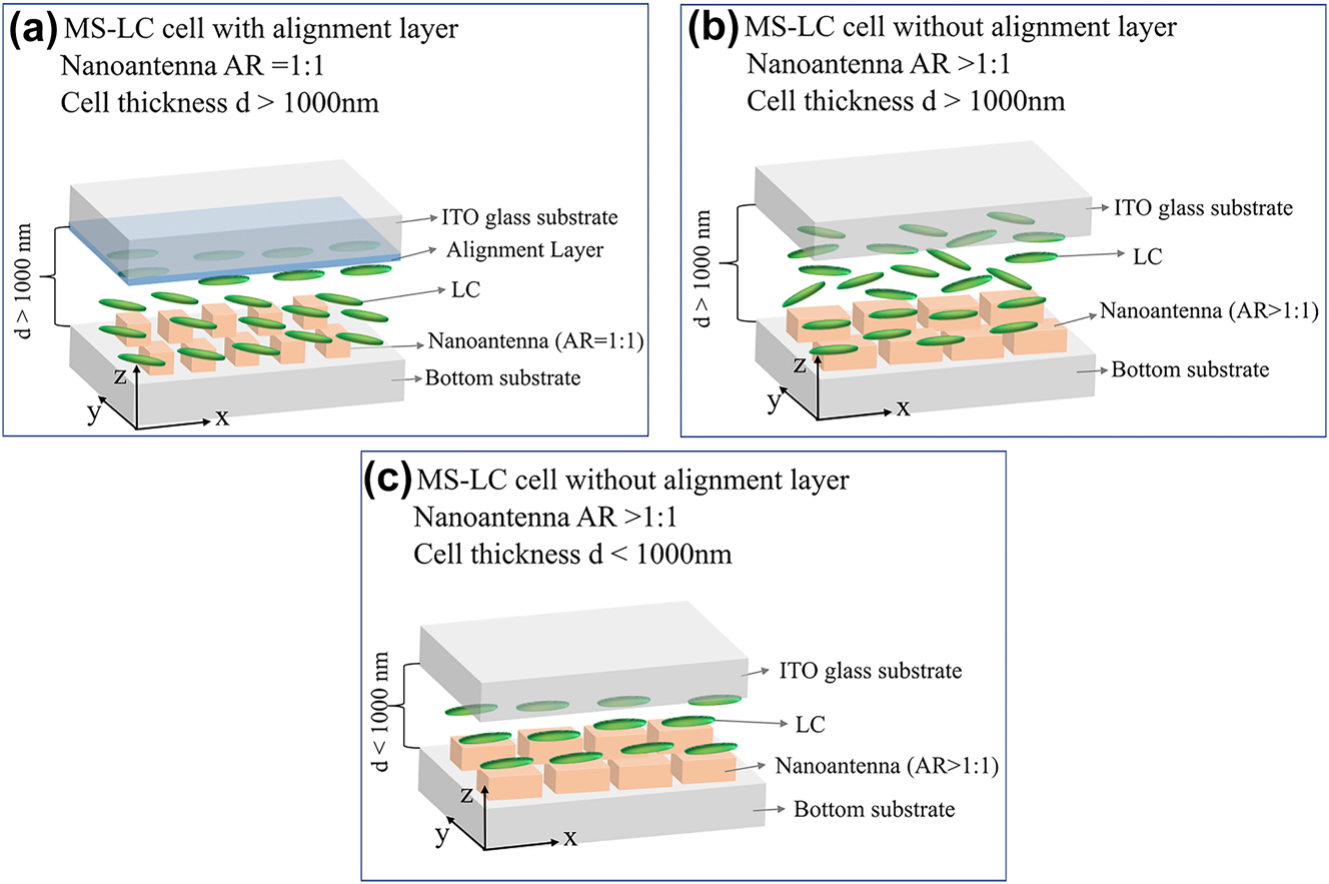
Metasurface-LC cell. (a) Schematic of the standard metasurface-liquid crystal (MS-LC) cell with alignment layer. The nanoantenna aspect ratio (AR) is 1:1 and the cell thickness is above 1000 nm. (b) Schematic of MS-LC cell with nanoantenna AR>1:1, cell thickness is above 1000 nm without an alignment layer. (c) Proposed MS-LC cell with nanoantenna AR>1:1 and cell thickness less than 1000 nm. Here, the LC alignment can be uniform and controlled by the nanoantenna geometry and periodicity without external alignment layer.

Figure 1(a)–(c) show the schematic view of a metasurface-LC (MS-LC) cell with different cell thickness range and anchoring conditions. Figure 1(a) is a “standard” (i.e., as previously reported in the literature [1], [2], [3, 30–35]) transmissive MS-LC cell in which the metasurface consists of nanoantennas with equal *x*:*y* aspect ratio (*xy*-AR = 1:1) and contains an alignment layer on the top electrode, whose function is to induce a preferential pre-alignment of the LC in the absence of bias [11, 22–24, 30–35, 44–48, 51–53, 61–63]. As reported in previous studies [30], this nanoantenna geometry induces an angular orientation for the LC that differs from that of the alignment layer. In the MS-LC cell as shown in Figure 1(b) and (c), the alignment layer is absent, and the nanoantennas have an aspect ratio in the *xy*-plane departing from 1:1 (*xy*-AR>1:1), which allows controlling the LC alignment in their vicinity. However, in Figure 1(b) the cell thickness is “large” (*d* > 1000 nm) and the absence of alignment layer results in nonuniform LC alignment across the cell height. As will be shown later and is schematically depicted in Figure 1(c), reducing the cell thickness (*d* < 1000 nm) allows maintaining the LC alignment induced by the nanoantenna geometry and periodicity across the cell, resulting in a uniform LC alignment. In all three cases, the device comprises a substrate and superstrate made of glass and coated with a thin (≈23 nm) transparent indium tin oxide (ITO) to form the electrodes and assembled to form the cell. As already mentioned, in the “standard” case the top electrode is coated with an alignment layer, which is typically either a polymer or a monomer, and which is from now on assumed to be treated to induce a preferential pre-alignment of the LC along the *x*-axis. There are two usual ways of controlling the direction of the alignment layer, which in turn control the pre-alignment direction of the LC director ( $\hat{n}$ , the average orientation of LC molecules). The first method is by irradiating a photosensitive material (for example azo-dye) with a polarized light source (typically in the UV spectral range), and hence known as photo-induced alignment. The second method is the mechanical rubbing of polyimide or polystyrene. The alignment direction is defined by the polarization of light in the first method and by the direction of rubbing in the second. For our “standard” devices (i.e., containing the alignment layer) we use the rubbing method for pre-alignment, as it is known to give more reliability than the photo-induced one (the full details on device fabrication are provided in the Supplementary Information).

In the nematic phase, the long axes of the LC molecules tend to align parallel to each other in a certain direction, the so-called director, which is apolar. The LC refractive indices parallel and perpendicular to the director are called the extraordinary refractive index (*n*_*e*_) and the ordinary refractive index (*n*_*o*_), respectively. The difference Δ*n* = *n*_*e*_ – *n*_*o*_ gives the birefringence (Δ*n*). The LC orientation is specified by two parameters: the polar angle (*θ*), and the azimuthal angle (*ϕ*) (see Figure S1 in the Supplementary Information). In the absence of electric field, the molecules are aligned in a specific direction defined by *θ* (measured with respect to the substrate normal) and *ϕ* (the in-plane rotation angle) defined by the anchoring conditions [3]. For the LC cell structure in Figure 1(a)–(c), the polar angle *θ* can vary from its initial, un-biased state *θ* = 90° (i.e., in-plane oriented, parallel to the substrate) to *θ* = 0° (i.e., out-of-plane oriented, perpendicular to the substrate) through electrical tuning. The azimuthal angle *ϕ* is usually controlled by the alignment layer.

To study the effect of the nanoantenna geometry and the cell thickness on the alignment performance of the LC in the metasurface, three different nanoantenna structures and three LC-cell thickness ranges are used. A cell thickness (d) in the range of *d* ≈ 500 nm is considered as ultra-thin, *d* ≈ 750 nm is a thin cell and *d* ≈ 1000 nm is considered as a thick cell. In all cases, the metasurfaces consist of a periodic array of nanoantennas made of TiO_2_, selected for its high refractive index (*n* ≈ 2.5) and low losses (*k* ≈ 0) across the visible spectrum. The geometrical parameters of the nanoantennas are such that they support optical resonances in this wavelength range, with both electric dipole (ED) and magnetic dipole (MD) modes excited. In previous studies, it has been shown that such nanoantennas can be used to induce controlled local phase and/or amplitude shifts to an incoming beam, and thus to dynamically manipulate its wavefront [30–35]. A commercial dual frequency liquid crystal (DFLC) DP002-016 (PhiChem-HCCH) with birefringence Δ*n* = 0.268 (*n*_*e*_ = 1.779, *n*_*o*_ = 1.511 at *λ* ≈ 589 nm and 20 °C, with negligible absorption) is used in our experiments. For the temperature range −20 °C–104 °C the DP002-016 LC is in nematic phase and exhibit dual frequency character (the experimental results described here are similar for other nematic LCs). The DFLC, which is infiltrated in the cell gap by capillary forces, is a LC mixture which exhibits positive dielectric anisotropy (Δ*ϵ* > 0) at lower driving bias frequency and a negative one (Δ*ϵ* < 0) above the cross over driving bias frequency (≈60 KHz). This allows the reorientation of its optical axis parallel to the electric field at low frequencies (<60 KHz) and perpendicular to it at high (>60 KHz) ones, for an applied voltage above the threshold [63]. The experiments are done for various anchoring strengths for comparison, a parameter that is controlled by the alignment layer rubbing, when it is present. All the measurements are done at room temperature.

**Figure 2:**
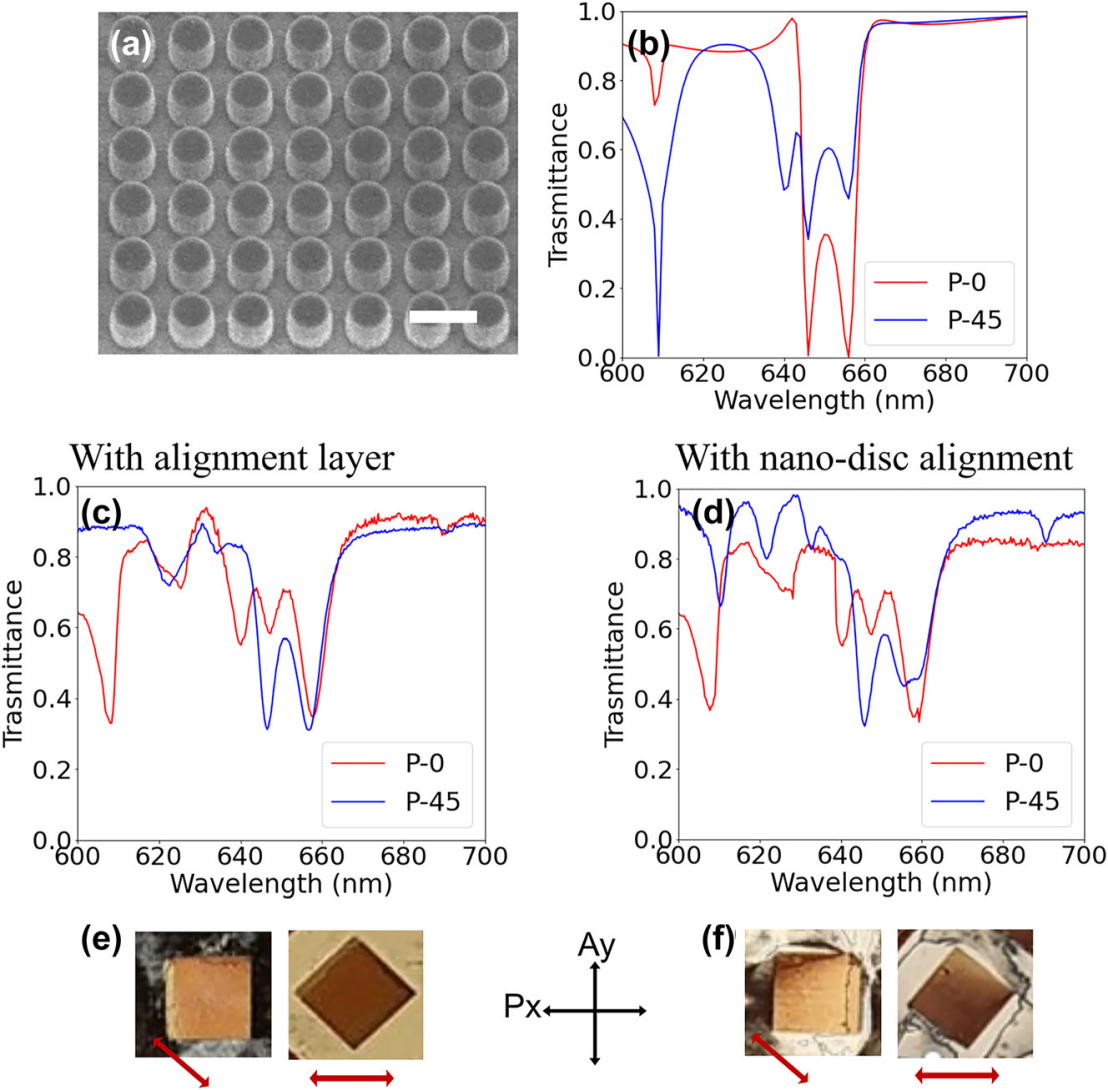
Nano-discs induced LC alignment. (a) The SEM image of the fabricated metasurface, the scale bar is 400 nm. (b) The calculated transmittance spectra of LC infiltrated nano-disc metasurface for LC layer thickness 750 nm for the incident polarization along *x*-axis (*P*-0) and 45° to the *x*-axis (*P*-45). (c), (d) The measured transmission spectra for nano-disc metasurface in a 750 nm thick LC cell for (c) with alignment layer and (d) pure nanoantenna induced alignment for incident light polarization *P*-0 and *P*-45. (e), (f) The images of LC infiltrated nano-disc metasurface array under crossed polarizer (*P*_*x*_) and analyzer (*A*_*y*_) for parallel and 45° orientations of array with respect to the *x*-axis. The metasurface array size is 100 × 100 μm. The red arrow indicates the LC alignment direction within the metasurface.

Figure 2(a) shows the scanning electron microscopy (SEM) image of the fabricated metasurface with the first geometry for the nanoantennas, namely, disc shaped particles with a diameter *D* = 270 nm, height *H* = 200 nm and periodicity (square lattice) *P* = 360 nm. This geometry, thus, has a 1:1 aspect ratio in the *xy*-plane (*xy*-AR). Due to the symmetric shape the ED and MD resonances excited by these nanoantennas are independent of the incident light polarization [32]. The optical transmission spectra of the fabricated bare metasurface (i.e. without LC) are provided in the Supplementary Information Figure S2(a) and associated discussion. Figure 2(b) shows the simulated transmission spectra of nano-disc MS-LC cell with thickness 750 nm for light polarization along the *x*-axis (*P*-0) and forming 45° with respect to the *x*-axis (*P*-45). The alignment is assumed in-plane, with the director along the *x*-axis (*θ* = 90° and *ϕ* = 0°). For *P*-0, the ED (at *λ* ≈ 646 nm) and the MD (at *λ* ≈ 657 nm) resonances are seen as partially overlapped dips, while the small narrow dip at *λ* ≈ 605 nm is an optical mode formed in the LC slab. For *P*-45, an additional resonance appears at ≈ 640 nm. Figure 2(c) and (d) show, respectively, the measured resonance spectra for MS-LC cells with polyimide alignment layer and for the pure metasurface induced alignment (i.e. without an alignment layer), for incident wave polarizations *P*-0 and *P*-45. The resonance spectra are measured using a customized inverted micro-spectrometry setup [30] (the details are described in the Materials and Methods section of the Supplementary Information). Upon close inspection and comparison with the simulation results of Figure 2(b), two things are noted. First, the resonance spectra measured from the cells with and without alignment layer show good correspondence with each other. This clearly indicates that the periodic arrangement of nanoantennas can, by itself and without the need of an alignment layer, induce a uniform alignment of the LC in the metasurface region for such thin cells. Second, the spectrum measured for the cells having an alignment layer and illuminated with an incident light with polarization 45° with respect to the *x*-axis (*P*-45) is matching with the simulation results for *P*-0. Vice versa, the measurement with *P*-0 is equivalent to the simulation for *P*-45. This seems to indicate that the metasurface induces an angular alignment with respect to the *x*-axis, even in the presence of an *x*-rubbed alignment layer. This stems from the symmetric structure of the nanoantennas and is in good agreement with previously reported results [30]. This angular alignment further indicates the strong anchoring influence of the nanostructure.

Figure 2(e) and (f) show the images of the LC infiltrated metasurface array with and without an alignment layer, respectively. The images are recorded under crossed polarizer (*P*_*x*_) and analyzer (*A*_*y*_) for the parallel and 45° orientation of the array with respect to the *x*-axis. The red arrow here indicates the deduced LC alignment direction within the metasurface. Indeed, in Figure 2(e), for the parallel orientation of the array, the background LC is dark (as the optical axis induced by the alignment layer is parallel to the polarizer), but the metasurface appears bright due to the nanoantenna induced 45° LC alignment. For the 45° orientation of metasurface array with respect to the *x*-axis, the background LC becomes bright, and the array turns dark. Similar alignment behavior is observed in Figure 2(f) for the cell without alignment layer (here the background LC does not follow any specific alignment due to the absence of alignment layer). The images confirm the uniform alignment of LC in the metasurface array for thin cells and the angular nature of this alignment. Figure S3 in the Supplementary Information gives the detailed comparison of simulated and measured spectra for three different conditions of LC infiltrated nano-disc metasurface without an alignment layer. For thick MS-LC cells a clear deviation is observed in the resonance spectra for cells with and without alignment, as shown in the Supplementary Information Figure S2. Indeed, for 1500 nm thick MS-LC cell, by looking at the spectra with (Figure S2(c)) and without alignment (Figure S2(d)), no apparent correlation exists between measured spectra. The microscope images for this “thick” LC cell corroborate the random alignment of LC within the metasurface in the absence of the alignment layer and the angular one, preferentially at 45° (or, equivalently, 135°) for the one having it.

The metasurface induced LC alignment for nanoantennas with *xy*-AR 1:1 is further tested using square shaped nanoantennas. The simulation and experimental results for this case are provided in the Supplementary Information (see Figure S4 and the corresponding discussion). For a thin LC cell (*d* < 1000 nm), this metasurface can, similarly to the nano-disc-shaped, induce a uniform LC molecular alignment without the need of any alignment layer. Also similarly, the metasurface induces an alignment with azimuthal angle (*ϕ*) of 45° (or 135°), as seen by the transmission spectrum measurement. This follows from the fact that, for the nanostructure with *xy*-AR 1:1 the anchoring energy is the same along *x* and *y* direction and thus the free energy minimization gives *ϕ* ≠ 0 LC alignment [30]. In this situation, the incident light polarization would need to be adjusted based on the initial orientation angle of LC, i.e., along the extraordinary refractive index, to achieve the maximum modulation upon application of electrical bias. As further seen in the experiments, for devices with symmetric nanoantennas, even the strong anchoring from the alignment layer on the top electrode does not help to remove the angular alignment. Instead, this might induce a hybrid alignment of LC in the metasurface LC cell as well as an increase in the threshold and switching voltages.

Two things can be concluded from these results. First, ultra-thin MS-LC cells can induce a homogeneous LC alignment without the need for standard LC alignment layers. Second, with nanoantennas with *xy*-AR ≈ 1:1 (and square lattices), only angular LC alignment is possible. This might be an issue or unwanted for certain applications that aim to maximize the modulation depth, since an azimuthal or hybrid alignment reduces the maximum effective refractive index tunability. In the following, we show that it is possible to overcome the angular alignment by using other nanoantenna designs, capable of inducing LC alignment along a designated direction, including *ϕ* = 0, while keeping the desired optical resonances necessary for light modulation.

**Figure 3:**
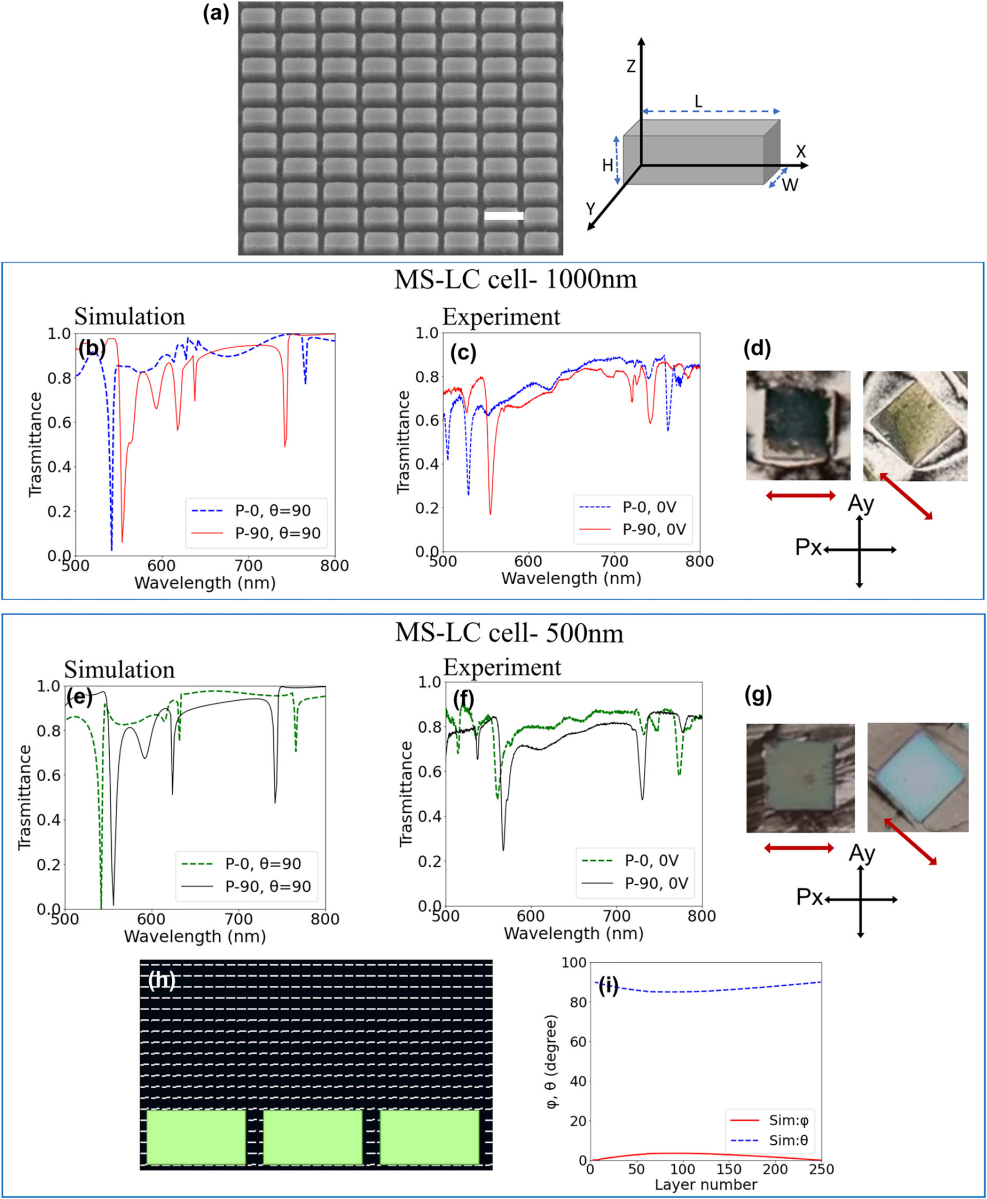
Nano-rectangle induced LC alignment. (a) SEM image of the fabricated nano-rectangle metasurface, the scale bar is 400 nm, and the schematic indicates the nano-rectangle orientation. The inset sketch shows the orientation of the nano-rectangle with respect to the *xy* coordinates. (b) Calculated and (c) measured transmittance spectra of the LC infiltrated nano-rectangle metasurface for a 1000 nm thick LC cell without alignment layer for incident light polarized along the *x*-axis (*P*-0, blue curve) and *y*-axis (*P*-90, red curve). (d) Microscope images of the LC infiltrated metasurface without top electrode alignment layer under crossed polarizer (*P*_*x*_) and analyzer (*A*_*y*_) for parallel and 45° orientation of metasurface array with respect to the *x*-axis. The metasurface is 100 × 100 μm in size. The red arrow indicates the LC director. (e) Calculated and (f) measured transmittance spectra of the nano-rectangle metasurface after LC infiltration for 500 nm thick cell for the incident light polarized in-plane along *x*-axis (*P*-0, green curve) and *y*-axis (*P*-90, black curve). (g) Microscope images of the LC infiltrated metasurface. (h) Simulated LC director profile in a nano-rectangle metasurface-LC cell. The director field is represented by the vectors in white. (i) Calculated layer-wise azimuthal (*ϕ*) and polar (*θ*) angles of LC in the nano-rectangle metasurface.

Our hypothesis is that, in an MS-LC device, to induce a parallel alignment of LC along designated direction, the nanoantennas must have a larger aspect ratio in the direction in which alignment is desired. In other words, the size of the nanostructures along the direction in which LC alignment is desired should be larger than its size along any other direction (e.g., if *x*-orientation is desired, an *xy*-AR: 2:1, 3:1, etc. would be necessary). For this purpose, one can use, e.g., a rectangular block or a cylinder with elliptical cross-section, among others. Here, we used metasurfaces comprising rectangular-shaped block nanoantennas. An additional means to induce LC alignment along a certain direction is by using periodic lattices with a longer lattice vector along the desired alignment direction. This can be achieved, e.g., using a rectangular lattice instead of a square one. Figure 3(a) shows an SEM image of a characteristic fabricated sample, where the long axis of the rectangular block nanoantenna is along the *x*-axis and has a length *L* = 360 nm. The width (length along the *y*-axis) is *W* = 200 nm, the height *H* = 200 nm and the lattice periods *P*_*x*_ = 420 nm and *P*_*y*_ = 280 nm.

Two cells, with thickness 1000 nm (“thick”) and 500 nm (“ultra-thin”), are fabricated without an alignment layer for the measurements. Their simulated transmission spectra, assuming a homogeneous (*θ* = 90, *ϕ* = 0) LC alignment, are shown in Figure 3(b) and (e), respectively, for incident polarizations *P*-0 and *P*-90. For the *P*-0 case, the MD and ED coincide at *λ* ≈ 770 nm, while the nanoantennas have higher order modes at *λ* ≈ 540 nm. When the polarization is *P*-90, one can see that the main mode at longer wavelengths blue-shifts. The dip at *λ* ≈ 620 nm is from an optical resonance in the LC slab. Figure 3(c) and (f) show the measured spectra for both MS-LC cells (the cells are fabricated without any alignment layer). Comparing the measured spectrum with the simulation, one can immediately see that metasurface can indeed provide homogeneous LC alignment along the desired direction, i.e., with angle *ϕ* nearly zero, as determined by the aspect ratio of the nanoantennas (namely its longest dimension) and the lattice periodicity. This is confirmed in Figure 3(d) and (g) showing the images of LC infiltrated nano-rectangle metasurface under crossed polarizer and analyzer for the thick and ultra-thin cells, respectively. Since the alignment induced by the rectangular nanoantenna is along the long dimension of the rectangle (along *x*-axis) the metasurface array appears dark for the *x*-axis orientation (LC director parallel to the polarizer) and bright (LC director 45° to the polarizer) for 45° rotation of the array with respect to the *x*-axis for both cells. The red arrow schematically indicates the deduced LC alignment direction within the metasurface. These images show the uniform parallel alignment of LC without any angular orientation. To further justify the experimental observations, Figure 3(h) shows the calculated director profile for the nano-rectangle metasurface-LC cell in a weak anchoring condition from the top substrate (corresponding to the absence of an alignment layer) for a unit cell with 3 nano-rectangles with periodicity in both *x*- and *y*-directions. The details of the LC director simulation and modelling are given in the Materials and Methods section of the Supplementary Information. Figure 3(i) is the calculated layer wise LC director angles *θ* and *ϕ* across the cell thickness, corroborating the planar alignment of LC across the cell, further confirming our experimental results. Transmission spectra of the nano-rectangle metasurface with the 500 nm-thick LC cell (without an alignment layer) under electrical biasing are shown in Figure S6 in the Supplementary Information. The results demonstrate that, under the applied electrical voltage, for the incident polarization *P*-0 the resonances have large displacement, while for *P*-90 case it is negligible. This further confirms the parallel alignment of LC director along the long axis of the rectangular nanoantenna without an alignment layer.

**Figure 4:**
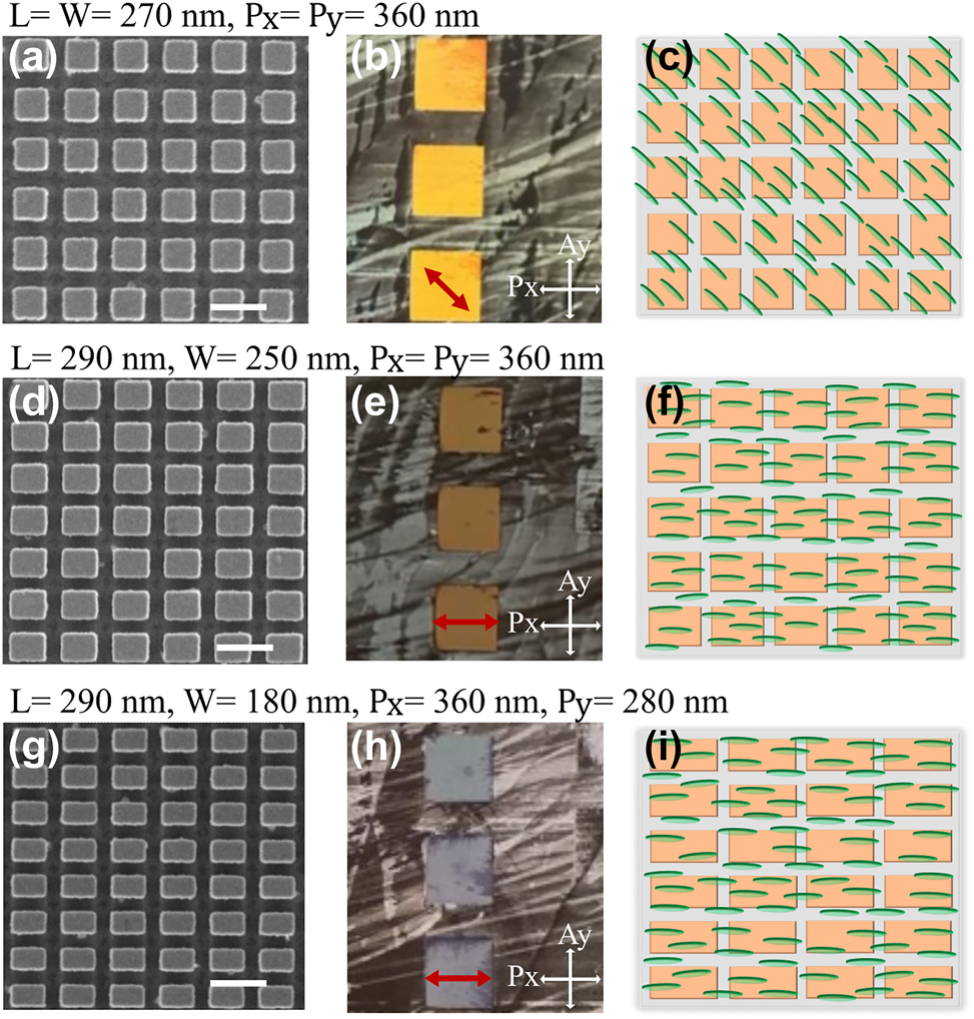
LC alignment for different nanoantenna aspect ratios. (a), (d), (g) SEM images of the fabricated nanoantenna metasurface. The scale bar is 400 nm. Optical microscope images of LC infiltrated metasurfaces consisting of (b) nano-squares with *L* = *W* = 260 nm, *H* = 200 nm, *P*_*x*_ = *P*_*y*_ = 360 nm, (e) nano-rectangle with *L* = 290 nm, *W* = 250 nm, *H* = 200 nm, *P*_*x*_ = *P*_*y*_ = 360 nm and (h) nano-rectangles with *L* = 290 nm, *W* = 180 nm, *H* = 200 nm, *P*_*x*_ = 360 nm, *P*_*y*_ = 280 nm in a 500 nm LC cell without an alignment layer. The images are recorded under crossed polarizer (*P*_*x*_) along the *x*-axis and analyzer (*A*_*y*_) along the *y*-axis. The metasurface array size is 100 × 100 μm. The red arrow indicates the LC director orientation in the metasurface. Figure S7 in the Supplementary Information shows the images of the same metasurfaces for different orientations of array with respect to the polarizer. The schematic shows the top view for the LC alignment within metasurface consisting of (c) square shaped symmetric nanoantenna with *x*:*y*-AR = 1:1 and (f), (i) asymmetric nanoantenna with AR> 1:1.

We should note at this point that the geometry of the nanoantennas can still be modified to generate different spectral responses (and resonances), as required for wavefront modulation, either based on amplitude and/or phase (including the commonly used Huygens’ condition for transmissive devices [64], while keeping an *xy* aspect ratio suitable for the desired LC alignment. Figure 4 shows that this is the case by analyzing the resulting LC alignment for different nanoantenna aspect ratios without an alignment layer. Figure 4(a–c) show the SEM images of the fabricated metasurfaces with different *xy*-ARs, ranging from square cross section (Figure 4(a)) with *L* = *W* = 270 nm, *P*_*x*_ = *P*_*y*_ = 360 nm to rectangular shapes (*L* = 290 nm, *W* = 250 nm, *P*_*x*_ = *P*_*y*_ = 360 nm for Figure 4(d), and *L* = 290 nm, *W* = 180 nm, *P*_*x*_ = 360 nm, *P*_*y*_ = 280 nm for Figure 4(g)). Figure 4(b), (e), (h) are the optical microscope images of the three sets of metasurface arrays under crossed polarizers with LC infiltration. All these nine metasurface arrays are fabricated on a single substrate with nanoantenna height *H* = 200 nm, and the LC cell is fabricated with thickness of 500 nm without an alignment layer. In Figure 4(e) and (h) the long axis of the nano-rectangles is oriented along the *x*-axis. The background LC alignment is nonuniform since there is no alignment layer. For the metasurface in Figure 4(a)–(c) both nanoantenna aspect ratio and the periodicity are symmetric, which leads to angular alignment, so the arrays in Figure 4(b) appear bright when they are parallel to the polarizer (LC director 45° to the polarizer as indicated by the red arrow). In Figure 4(d)–(f) the nanoantenna aspect ratio *xy*-AR> 1:1, but the metasurface maintains the lattice symmetry, while in Figure 4(g–i) both the antenna dimensions and the periodicity are asymmetric. In both cases, the arrays appear dark when they are parallel to the polarizer since the LC director is also parallel to the polarizer. Thus, these nanoantenna with aspect ratio *xy*-AR> 1:1 allow controlled LC alignment in the desired direction, which is along the long axis of the nano-rectangle here. Figure S8 in the Supplementary Information shows the simulated transmission spectrum and the corresponding phase shift for one of the rectangular nano-blocks (*L* = 290 nm, *W* = 250 nm, *P*_*x*_ = *P*_*y*_ = 360 nm), in a 500 nm thick LC cell, for homogeneous and homeotropic alignment of LC. For the homogeneous LC alignment at *λ* ≈ 670 nm, the ED and MD resonance are overlapped with an associated ≈2*π* phase modulation. The simulation and experimental observations confirm that for the miniature MS-LC devices, the nanoantennas can be designed for desired wavefront control, while acting as alignment layer for the LC.

**Figure 5:**
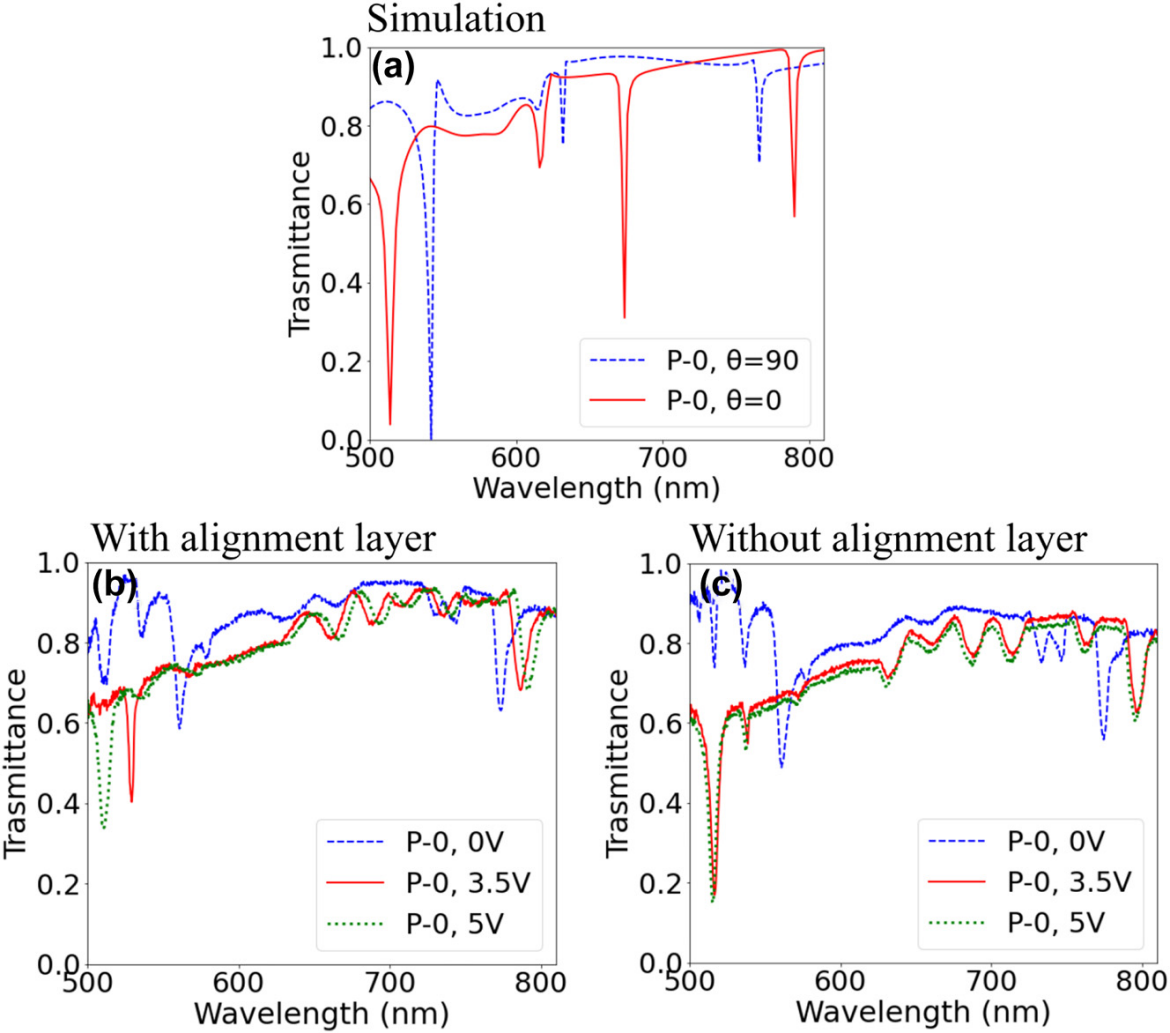
Nano-rectangle metasurface resonance tuning. (a) Calculated transmission spectra for the LC infiltrated nano-rectangle MS for homogeneous (*θ* = 90, blue curve) and homeotropic (*θ* = 0, red curve) LC alignment. The incident light polarization is along the *x*-axis (*P*-0). The measured transmission spectra of the MS-LC cell (b) with top electrode alignment layer and (c) with pure metasurface induced alignment for the applied voltages of 0 *V*_rms_ (blue curve), 3.5 *V*_rms_ (red curve) and 5 *V*_rms_ (green dotted curve) for the incident light polarization along the *x*-axis (*P*-0).

Since the ultra-thin MS-LC cell with nanoantenna induced alignment does not include any additional alignment layer, which is known to strongly affect the LC behavior in its surrounding, the voltage level required for light modulation is expected to be reduced and the response speed (and thus potentially the refresh rate) increased compared to a device with the alignment layer. To demonstrate that we first focus on the voltage characteristics. Figure 5(a) shows the calculated transmission spectra of the LC-infiltrated nano-rectangle metasurface (*L* = 360 nm, *W* = 200 nm, *H* = 200 nm, *P*_*x*_ = 420 nm and *P*_*y*_ = 280 nm) for homogeneous (*θ* = 90°) and homeotropic (*θ* = 0°) LC alignment. Figure 5(b) and (c) show the electrical tuning of rectangular nanoantenna resonances for MS-LC cells with the same (500 nm) cell thickness in two cases: a cell with top electrode alignment layer (Figure 5(b)) and for metasurface induced alignment (Figure 5(c)). Comparing the resonance shift with respect to the applied voltage, it becomes apparent that the cell with metasurface-induced alignment requires lower voltage than the cell with the alignment layer. Indeed, for the latter case (Figure 5(c)), the spectral position of the resonances is already saturated for 3.5*V*_rms_, (no further shift is observed upon applying voltage above 3.5*V*_rms_), which is not the case for the cell containing the alignment layer (Figure 5(b)), where the resonance at ≈530 nm is still far from its saturation position for 3.5*V*_rms_. This is further corroborated by the optical images of the samples under crossed polarizer and analyzer for different applied voltage, as shown in Figure S9 (the metasurfaces here are oriented at 45° with respect to the polarizer). The threshold voltage required for the LC switching in the cell with metasurface induced alignment is in the range of 0.6 to 0.8*V*_rms_, while that of the cell with the alignment layer needs a minimum of 1.7*V*_rms_. Thus, for the cell with pure metasurface induced alignment the threshold voltage is reduced to less than a half, and the saturation voltage is reduced by 30 % (from 5*V*_rms_ to a value of 3.5*V*_rms_).

**Figure 6:**
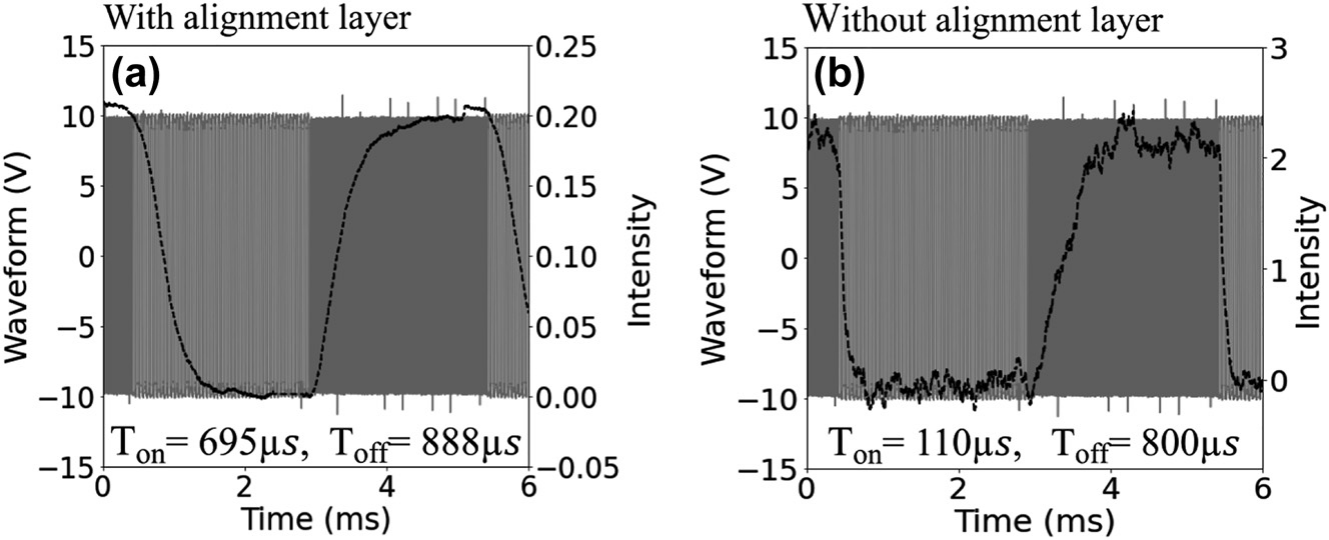
Response time *T*_on_ and *T*_off_ for nano-rectangle MS-LC cell. The switching performance of the sample (voltage dependent transmitted intensity-black curve) and the applied signals (gray) for a 750 nm cell (a) with top electrode alignment layer (*T*_on_ = 695 μs, *T*_off_ = 888 μs) and (b) without the alignment layer (*T*_on_ = 110 μs, *T*_off_ = 800 μs). The dual-frequency pulse is switched between *f*_low_ = 20 KHz and *f*_high_ = 200 KHz for applied voltage 10*V*_pp_.

Another driving parameter, the response time, also depends on the anchoring strength and the cell thickness. The total response time is usually referred to the sum of the rise and decay time [23, 24]. We measured the response time (see Materials and Methods in the Supplementary Information) of two different thin-LC (*d* = 750 nm) cell configurations (with and without the alignment layer) for the nano-rectangle (*L* = 360 nm, *W* = 200 nm, *H* = 200 nm, *P*_*x*_ = 420 nm and *P*_*y*_ = 280 nm) metasurface. For that, a dual-frequency control pulse is applied to the DFLC infiltrated device. The measurement results are shown in Figure 6. The low (20 KHz) and the high (200 KHz) frequency signal sections have the same duration. For the MS-LC cell without alignment, the switch-on speed is much faster than that for the cell with the alignment layer (110 μs vs 695 μs, respectively), which results in ≈6× increase in speed. On the other hand, the ‘off’ time is almost the same for both types of the cells (800 μs), making the total response time of the cell without the alignment layer below 1 ms. Compared with the DC balanced driving method, the total response time is shortened for dual frequency (DF) switching method, since both “on” and “off” responses are voltage driven. The difference between the “on” and “off” times is due to the difference in the dielectric anisotropy at 20 kHz (Δ*ϵ* = 4.7) and 200 kHz (Δ*ϵ* = −2.7). They can be further equalized to a few hundred microseconds each by optimizing the voltage and frequency combination of the applied signal. For completeness, we show in Figure S10 in the Supplementary Information the switching performance of the same cells under DC balanced driving for an applied peak to peak voltage of 10*V*_pp_ at 1 KHz (Δ*ϵ* = 9.8). Here, only switch-on time is controlled by the voltage, while the switch-off time depends on the relaxation of the LC molecules, which is slower.

## 3 Conclusions

We propose a new LC alignment strategy for LC-tunable metasurfaces and spatial light modulator devices. In the proposed method, the metasurface, consisting of sub-wavelength resonant nanoantennas, serves the double purpose of, first, in combination with ultra-thin LC cells, creating a uniform LC alignment without the need of any alignment layers and, second, serving as means for efficient light intensity and/or wavefront modulation. We further show that, for commonly used square nanoantenna lattices with nanoantenna *xy* aspect ratios 1:1, the resulting induced alignment, despite homogeneous, is diagonal to the array, even in the presence of an alignment layer. This indicates the strong anchoring induced by the nanoantenna structural geometry. To solve this issue, we introduce here a solution consisting of increasing the aspect ratio of the nanoantenna/lattice along the desired alignment direction. We show that this nanoantenna geometry induces homogeneous alignment of LC molecules along the long axis of the nanoantenna/period, even without an alignment layer. The absence of alignment layer allows to bring the threshold voltage down to less than half compared to the case with alignment layer (to a value of only 0.6 *V*_rms_), to reduce the saturation voltage by 30 % (to a value of 3.5*V*_rms_) and to reduce the total response time by ≈ 2× (with > 6× reduction of the switch-on time, bringing the total time to less than 1 ms using dual frequency driving). Also, the removal of the alignment layer simplifies the device fabrication and may help to increase the device lifetime by eliminating the light and heat induced degradation of alignment layer, and hence the LC quality. The proposed method thus allows light-modulating devices with low voltage, sub-millisecond switching, and pixel-size miniaturization by reducing the LC thickness, with wide applications in augmented and virtual reality displays, solid-state light detection and ranging (LiDAR), and 3D holographic displays, to mention some. While studied here in detail using TiO_2_ as the material platform, preliminary results not shown in this work seem to indicate that this strategy might also be effective for another commonly used material, namely silicon. Nevertheless, its broader applicability to other materials will certainly require additional exploration. Similarly, extending this concept to other, more complex, metasurface geometries (e.g. metalenses), while in principle possible will not be straightforward and possibly demand additional developments of the method.

## Supplementary Material

Supplementary Material Details
