# Supplementary material for: Nanoantenna induced liquid crystal alignment for high performance tunable metasurface
Source: Nanophotonics. 2023 Sep 26;13(12):2127–39. doi: 10.1515/nanoph-2023-0446 (PMC11501184; doi:10.1515/nanoph-2023-0446)
Supplement: Supplementary file 1 — Supplementary Material Details [file j_nanoph-2023-0446_suppl_001.pdf]

Supplementary Material

Nanoantenna Induced Liquid Crystal Alignment for High Performance Tunable Metasurface

Rasna Maruthiyodan Veetil[1*], Xuewu Xu[1], Jayasri Dontabhaktuni[2], Xinan Liang[1], Arseniy I. Kuznetsov[1*], Ramon Paniagua-Dominguez[1*].

[1]Institute of Materials Research and Engineering (IMRE), Agency for Science, Technology and Research (A*STAR), 2 Fusionopolis Way, Innovis #08-03, Singapore 138634, Republic of Singapore.

[2] Department of Physics, Ecole Centrale School of Engineering, Mahindra University, Hyderabad 500 043, India.

*Email: ramon_paniagua@imre.a-star.edu.sg

*Email: arseniy_kuznetsov@imre.a-star.edu.sg

* Email: Rasna_Maruthiyodan_Veetil@imre.a-star.edu.sg

MATERIALS AND METHODS

Metasurface Fabrication:

The metasurface is fabricated on an indium tin oxide (ITO) coated conductive glass slide (Latech Scientific Supply Pte. Ltd, Singapore) with ITO film thickness of 23 ± 5 nm. Amorphous TiO_2 layer of thickness 200 nm is deposited on the ITO glass slide by ion-assisted deposition system (Oxford Optofab 3000) followed by a 30nm thick layer of chromium (Cr) deposited by evaporation (Evovac, Angstrom Engineering). To pattern the nanoantennas, electron-beam lithography (Elionix ELS-7000) is performed using negative tone electron-beam resist, hydrogen silsesquioxane (HSQ, Fox-22, Dow-Corning Inc.). The pattern is transferred to the Cr layer by a reactive ion etching process in Oxford PlasmaLab 100. Using the Cr as a hard mask, TiO_2 nanoantenna is patterned through dry etching with CHF_3 gas. The chromium etchant solution (Sigma-Aldrich) is used to remove the Cr mask. Scanning electron microscopy (SEM) imaging of the fabricated TiO_2 nanoantenna is performed on Hitachi SU8200 ultra-high resolution scanning electron microscope.

Liquid Crystal (LC) Cell Fabrication:

The LC cell is fabricated by assembling two ITO conductive glass substrates. The ITO glass with metasurface pattern acts as a bottom substrate. For the cell with alignment layer, the top ITO electrode substrate is spin coated with a polyimide and cured at 150°C for 30 minutes. Substrate is then rubbed unidirectionally using a commercial rubbing machine (Holmarc Opto-Mechatronics HO-IAD-BTR-03). The rubbing direction and strength defines the orientation of LC molecules and their anchoring energy. For the cell without alignment layer, a clean ITO glass without any treatment is used as a top substrate electrode. The top substrate is pressed on the metasurface patterned bottom substrate with the help of a homemade

press, containing multi-point optical profilometer. The UV-curable adhesive (Norland optical adhesive NOA81) acts as a spacer and keeps the substrates attached. The thickness of the empty cell is determined using the spectrum measured with Ocean Optics spectrometer (HR 4000CG-UV-NIR) and spectra-suit software. Dual frequency LC is encapsulated in the cell through capillary filling.

Optical Simulations:

The optical simulations are performed using COMSOL Multiphysics software package. One single unit-cell containing a disc or square or rectangular TiO_2 nanoantenna is considered, to which periodic boundary conditions (both the *x*- and *y*-directions) are applied. The top and bottom glasses are modelled as semi-infinite media using ports (also used to excite the system with a normally-incident plane wave) and the ITO layer (20 nm) is included in the simulations. The LC is modelled as an anisotropic medium defined by its dielectric permittivity tensor.

Optical Characterization:

The metasurface-LC cell is illuminated using a halogen lamp, focused through 20x Nikon microscope objective lens, giving a close to normal incidence. Incident light is linearly polarized with the desired orientation using a rotatable wire-grid polarizer (Thorlabs Visible Wire Grid Polarizers, extinction ratio above 800:1 within the 420~700 nm wavelength range). The transmittance is obtained by normalizing the transmission intensity to that of the light source, both detected using a spectrometer (Andor Kymera 328i) with a 400×1600-pixel EMCCD (Andor Newton) attached to the microscope setup.

LC Modelling and Simulation:

We have considered a periodic array of nano-disc of dimensions D= 270 nm, H= 200 nm and nano-rectangles of dimensions L = 370 nm, W= 200 nm, H=200 nm respectively, inside an LC cell compatible with the experimental conditions. The unit cell consists of three nanostructures with periodicities along *x*- and *y*-direction with P_x=420 nm and P_y=280 nm for the case of nano rectangles and P_x=P_y= 360 nm for nano-discs. The free energy of nematic LC medium is written in terms of order parameter tensor, Q_{ij} according to the Landau-de Gennes formalism:

$$f = \frac{1}{2}a(T - T *_{NI})Q_{ij}Q_{ji} + \frac{1}{3}BQ_{ij}Q_{jk}Q_{ki} + \frac{1}{4}C(Q_{ij}Q_{ji})^2 + \frac{1}{2}W(Q_{ij} - Q_{ij}^0)^2 + \frac{1}{2}K\frac{\partial Q_{ij}}{\partial x_k}\frac{\partial Q_{ij}}{\partial x_k}$$

where, a, B and C are temperature dependent constants, T_{NI}^* represents nematic-isotropic transition temperature, W is the surface anchoring strength, Q_{ij}^0 represents the surface-preferred order and K represents the Frank's single elastic constant ($K_{11} = K_{22} = K_{33}$). The total free energy is minimised using explicit finite difference scheme employing Euler-Lagrangian formalism to obtain stable configurations.

In the simulations, surface anchoring at the top and bottom substrates is considered to be weak planar ($W = 10^{-5}$ N/m^2) and the anchoring imposed by the surfaces of the nano antennas is considered to be weak in random orientations ($W = 10^{-5}$ N/m^2).

Response Time Measurement:

The response time of the device under two types of driving scheme, such as Frequency-Shift Keying (FSK) scheme and DC balanced scheme, is measured with a traditional cross polarization optical setup. For the FSK driving scheme, the driving signal hops between two driving signals of different frequencies, and the hopping rate can be set as desired. The DC balanced driving scheme is similar to a normal square waveform

driving signal. In the optical setup, a collimated fiber laser beam with a wavelength of 650nm coming from a supercontinuum source (SuperK EXTREME, NKT Photonics) equipped with multi-wavelength filter (SuperK SELECT, NKT Photonics) is used as the illumination source. The metasurface-LC cell is placed between the cross polarizer and analyzer, with LC alignment direction 45° with respect to the polarizer. The collimated laser beam is normally incident to and passes through the polarizer, cell, and analyzer sequentially and is detected finally with a high-speed silicon photodetector (Thorlabs PDA36A2). The photodetector is then connected to a digital oscilloscope (Rohde & Schwarz RTB2004 2.5GSa/s) to record the response of the LC cell upon applying the driving signal generated with the function generator. The response curve recorded with the oscilloscope is then used to calculate the rising and falling time of the device. The time difference between 10% and 90% of the total intensity change is used as the time constant of the device.

RESULTS

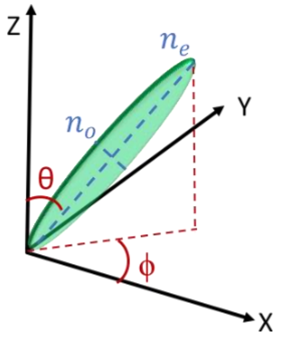

Fig. S1. A sketch of an LC molecular orientation: The polar angle θ and azimuth angle ϕ define the average orientation of LC molecules.

Nano-disc induced LC alignment for thick cells:

Figure S2(a) shows the simulated and measured optical transmission spectra for nano-disc metasurface before LC infiltration. Good correspondence is found between full numerical simulations and the optical transmission spectra of the fabricated sample. In figure S2(a) the resonance dips at λ≈ 550 nm and λ≈ 600 nm correspond to the excitation of ED and MD modes, respectively, supported by the nano-discs. Due to the symmetric shape of the nanoantennas, the resonances are independent of the incident light polarization direction. Figure S2(b) shows the simulated transmission spectra of nano-disc MS-LC cell with cell thickness 1500 nm for light polarization along the *x*-axis (P-0) and 45° with respect to the *x*-axis (P-45). For P-0, the ED (at λ≈ 646 nm) and the MD (at λ≈ 657 nm) resonances are seen as partially overlapped dips, while the small narrow dip at λ≈ 605 nm is a cavity mode formed in the slab. For P-45, the signatures of different multipole resonances appear. Figures S2(c) and S2(d) show the measured resonance spectra for incident wave polarization P-0 and P-45for 1500 nm thick LC cell with and without alignment layer. The rubbing direction, and hence the LC orientation induced by the alignment layer in the case when it is present, is along the *x*-axis. Figures S2(e) and S2(f) show the images of the LC infiltrated metasurface under crossed polarizer (P_x) and analyzer (A_y) for the parallel and 45° orientation of metasurface array with respect to the *x*-axis. The images clearly show, for this "thick" LC cell, the misalignment of LC within the metasurface in the absence of the alignment layer. This is corroborated by looking at the measured spectra with and

without alignment, where a clear deviation between the two is apparent. Also, the spectrum measured for the cells having an alignment layer and illuminated with an incident light with polarization 45° with respect to the *x*-axis (P-45) is matching with the simulation results for P-0. Vice versa, the measurement with P-0 is equivalent to the simulation for P-45. This indicates that the metasurface induces an angular alignment with respect to the *x*-axis, even in the presence of an *x*-rubbed alignment layer.

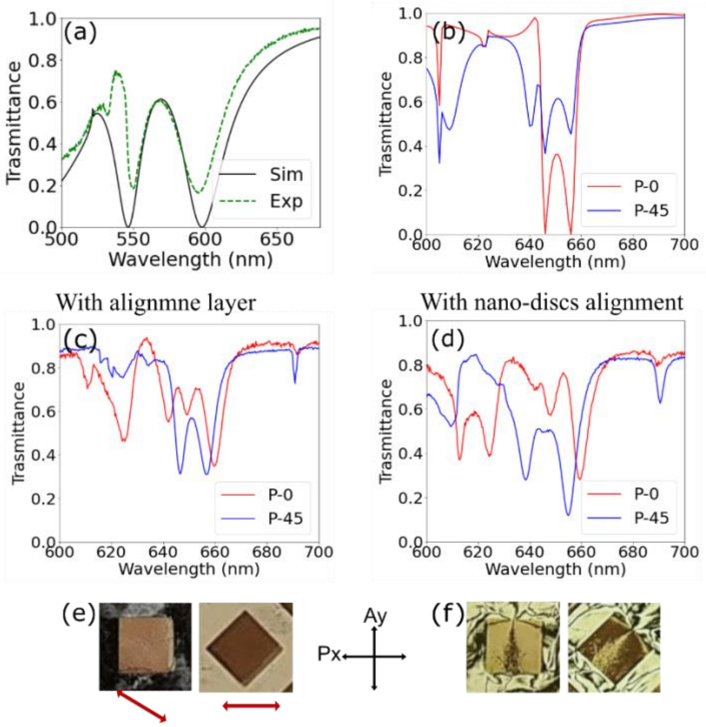

Fig. S2. Nano-discs induced LC alignment: (a) Calculated (black curve) and measured (green curve) transmission spectra of the nano-disc metasurface before the LC infiltration for incident light polarization along x-axis (P-0). (b) The calculated transmittance spectra of LC infiltrated nano-disc metasurface for LC layer thickness 1500 nm for the incident polarization along x-axis (P-0) and 45°to the x-axis (P-45). (c), (d) The measured transmission spectra for nano-disc metasurface in a 1500 nm thick LC cell for (c) with alignment layer and (d) pure nanoantenna induced alignment for incident light polarization P-0 and P-45. (e), (f) The images of LC infiltrated nano-disc metasurface array under crossed polarizer (P_x) and analyzer (A_y) for parallel and 45° orientations of array with respect to the x-axis. The metasurface array size is 100×100 μm. The red arrow indicates the LC alignment direction within the metasurface.

Nano-disc metasurface resonance tuning:

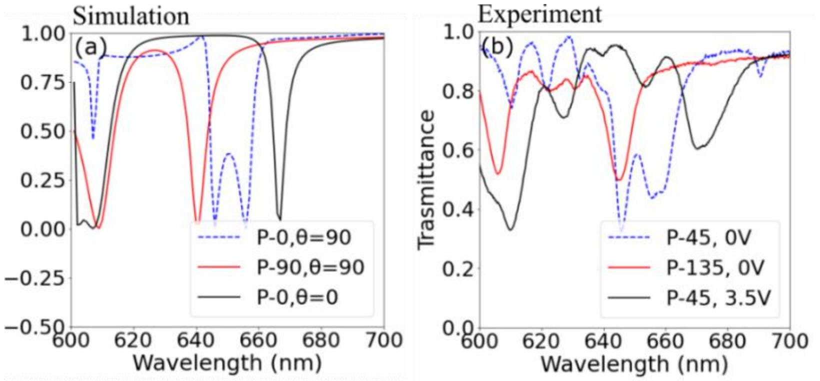

Fig. S3. LC tunable nano-disc metasurface; (a) Calculated transmission spectra of LC infiltrated nano-disc metasurface with homogeneous LC alignment (θ = 90) for light polarization along the x- axis (P-0, blue dashed curve) and y-axis (P-90, red curve). Black curve is for the homeotropic LC alignment (θ = 0) for the light polarization along x- axis (P-0). (b) The measured transmittance spectra for LC infiltrated nano-disc metasurface in 750 nm thick LC cell for the incident polarization 45° to the x-axis (P-45, blue dashed curve) and 135° to the x-axis (P-135, red curve) at 0 V_{rms}. Black curve is for an applied voltage of 3.5 V_{rms}.

Nano-squares metasurface induced LC alignment:

The metasurface induced LC alignment is verified using square shaped nanoantenna. Similar to the nano-disc, the nano-square also has aspect ratio (AR) 1:1. Figure S4(a) shows the scanning electron microscopy (SEM) images of the fabricated nano-square metasurfaces. Figure S4(b) shows the simulated and measured optical transmission spectra of metasurfaces comprising square nanoantennas with 1:1 aspect ratio in the xy plane (length L= 270 nm, width W= 270 nm) before LC infiltration. The antenna height is H= 200 nm and the periodicity P= 360 nm. Compared to nano-discs, the resonances are slightly shifted (λ≈ 575 nm and λ≈ 620 nm) as expected for a particle with slightly larger total volume. Due to the symmetric shape of the nanoantennas, the resonance is independent of the incident light polarization direction. Figure S4 (c) shows the simulated transmission spectrum for LC infiltrated nano-square metasurface with cell thickness of 750 nm, for different LC alignment and incident light polarization conditions. For the homogeneous alignment of LC (θ= 90), for incident polarization P-0, the dip observed at λ≈ 668 nm corresponds to electric and magnetic dipole resonances excited in the nanoantennas. For the homeotropic alignment of LC (θ= 0), the molecules are aligned vertically (which would correspond to the situation in which a bias, above the saturation value is applied between the top and bottom electrodes) and the resonances red shift to λ≈ 682 nm while higher order modes are excited at λ≈ 628 nm. The dip at λ≈ 603 nm is a cavity mode formed in the LC slab. Figures S4(d) and figure S4(e) show the measured transmission spectrum for 750 nm thick metasurface-LC cell with an alignment layer present in the top electrode (rubbing direction along x) and the device with pure metasurface induced alignment, respectively, for different applied voltages and incident polarization condition. Comparing the measured spectrum with the simulation, for thin LC cell (here 750 nm), the metasurface can induce a uniform LC molecular alignment without need of any alignment layer. However, for these nanoantennas with AR~ 1:1, the metasurface induces an alignment with azimuth angle (ϕ) of 45^0 or 135°, as seen by the transmission measurement. Figures S4(f) and S4(g) show the images of LC infiltrated nano-square metasurface under crossed polarizer and analyzer for parallel and 45° orientation of metasurface array with respect to the polarizer. Figure S4(f) is for the cell with alignment layer and Figure S4(g) for the cell with pure metasurface induced alignment. The image confirms the uniform and angular alignment of LC in the cells with nano-square metasurface.

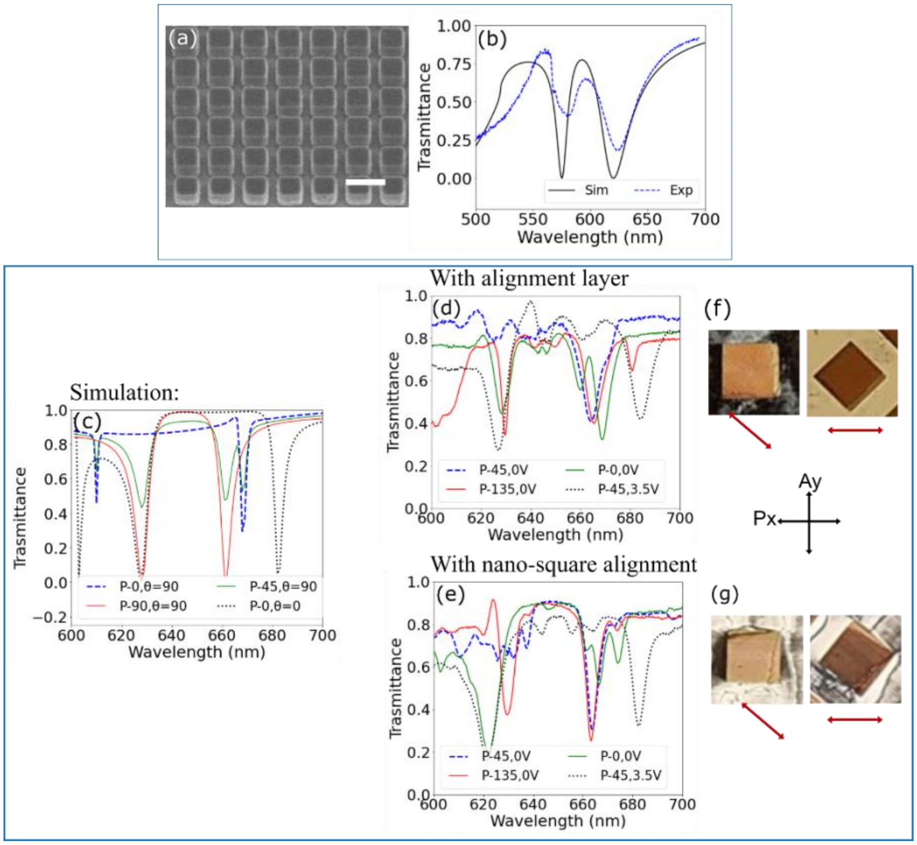

Fig. S4. Nano-square induced LC alignment: (a) The SEM image of the fabricated metasurface, the scale bar is 400 nm. (b) Calculated (black curve) and measured (blue dashed curve) transmission spectra of the nano-squares (L=W=270nm, H=200nm, P=360nm) metasurface before the LC infiltration. (c) Calculated transmission spectra of the LC infiltrated nano-square metasurface for homogeneous (θ= 90, blue dashed curve) and homeotropic (θ= 0, black dotted curve) alignment of LC for light polarization along the x-axis (P-0). The red and green curves are for polarizations P-90 and P-45, respectively, with respect to the x-axis. (d) The measured transmission spectra for 750nm thick metasurface-LC cell with top electrode alignment layer for applied voltage of 0 V_{rms} (blue dashed curve) and 3.5 V_{rms} (black dotted curve) for incident light polarization P-45. (e) The measured transmission spectra for 750 nm thick nano-square MS-LC cell without an alignment layer for applied voltage of 0 V_{rms} (blue dashed curve) and 3.5 V_{rms} (black dotted curve) for incident light polarization P-45. The red and green curves are for incident light polarization P-135 and P-0, respectively, at 0 V_{rms}. (f) and (g) The images of LC infiltrated nano-square metasurface under crossed polarizer (P_x) and analyzer (A_y) for parallel and 45° orientation of the array with respect to the x-axis with and without alignment layer, respectively. The array size is 100×100 µm. The red arrow indicates the LC director orientation within the metasurface.

Nano-rectangle induced LC alignment and the resonance tuning:

Figures S5(a) and S5(b) show the calculated and measured transmittance spectra for light polarized along the *x*-axis (P-0) and *y*-axis (P-90) before LC infiltration. For incident polarization P-0, at λ ≈ 700 nm, the MD and ED resonances overlap, and for P-90 the resonances blue shift to λ ≈ 665 nm. Figure S6 shows the transmission spectra of the nano-rectangle metasurface with the 500 nm-thick LC cell (without an alignment layer) under electrical biasing. The results demonstrate that, under the applied electrical voltage, for the

incident polarization P-0 the resonances have large displacement, while for P-90 case it is negligible. This confirms the parallel alignment of LC director along the long axis of the rectangular nanoantenna without an alignment layer.

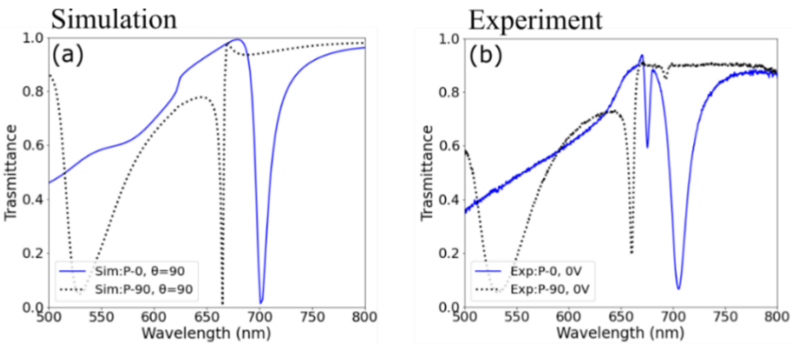

Fig. S5: (a) Calculated and (b) measured transmittance spectra of the nano-rectangle metasurface before LC infiltration for polarization along the x-axis (P-0, blue curve) and y-axis (P-90, black curve).

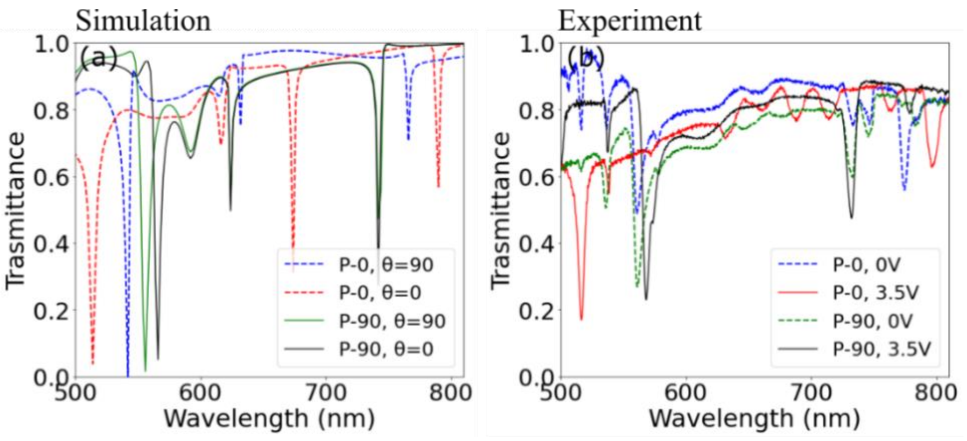

Fig. S6 Resonance tuning of nano-rectangle metasurface: (a) Calculated transmission spectra for LC infiltrated nano-rectangle metasurface for incident light polarization along the x-axis (P-0) for homogeneous (θ=90, blue curve) and homeotropic (θ=0, red curve) LC alignment. Green and black curves are for light polarization along the y-axis (P-90), for θ= 90 and θ= 0, respectively. (b) Measured transmission spectra for nano-rectangle MS-LC cell for incident light polarization along x-axis (P-0), at 0 V_{rms} (blue curve) and 3.5 V_{rms} (red curve). Green and black curves for incident light polarization along y-axis (P-90) at 0 V_{rms} and 3.5 V_{rms} ,respectively. The LC cell thickness is 500 nm.

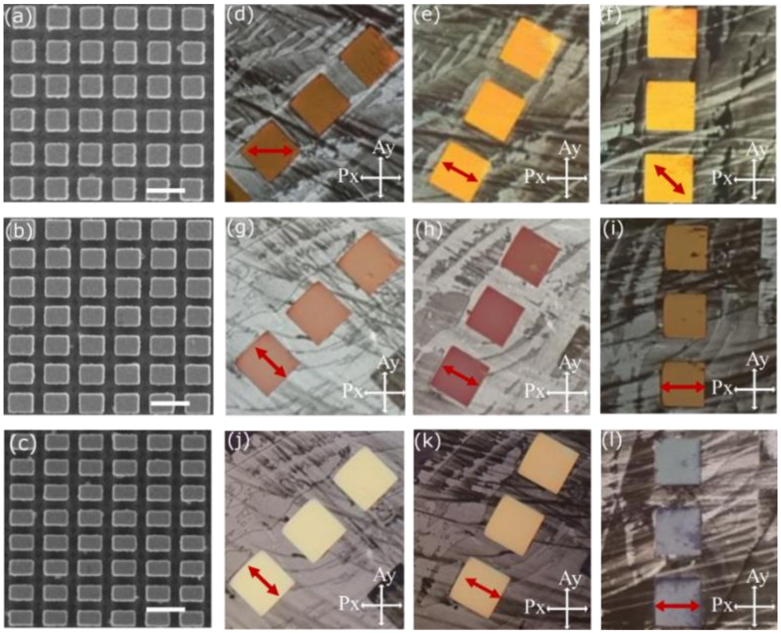

Fig. S7: (a)-(c) SEM images of the fabricated nanoantenna metasurface arrays, the scale bar is 400 nm. Microscope images of LC infiltrated metasurface consisting of (d-f) nano-squares with L= W= 260 nm, H= 200 nm, $P_x = P_y$= 360 nm, (g-i) nano-rectangle with L= 290 nm, W= 250 nm, H= 200 nm, P_x= P_y= 360 nm and (j-l) nano-rectangles with L= 290 nm, W= 180 nm, H= 200 nm, P_x= 360 nm, P_y= 280 nm under crossed polarizer (P_x) and analyzer (A_y) in a 500 nm LC cell without alignment layer for different array orientations.. The metasurface array size is 100×100 µm. The red arrow indicates the LC director orientation in the metasurface.

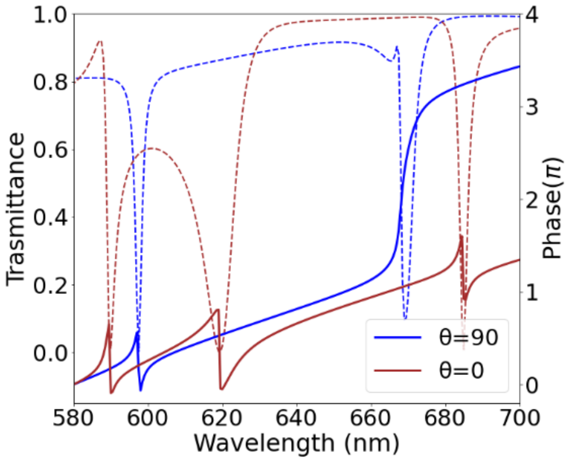

Fig. S8 Transmittance and phase tuning by nano-rectangle metasurface: Simulated transmission (dotted curve) and phase shifts (continuous line) for DFLC infiltrated nano-rectangle metasurface for the homogeneous (θ=90, blue curves) and homeotropic (θ=0, brown curves) alignment of LC in a 500nm thick cell.

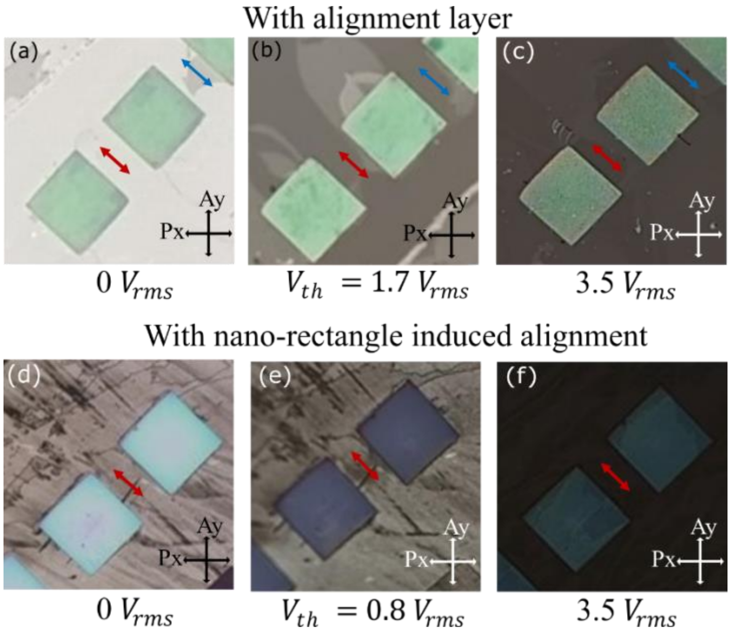

Fig. S9. Electrical switching of nano-rectangle metasurface: Images of LC infiltrated nano-rectangle metasurface under crossed polarizer (P_x) and analyzer (A_y) for different applied voltages for 500nm thick LC cells (a)-(c) with top electrode alignment layer, (d)-(f) with pure metasurface induced LC alignment without an alignment layer. V_{th} indicates the threshold voltage. The metasurface array is 100×100 μm in size. The red arrow indicates the LC director, and the blue arrow shows the top electrode induced alignment direction.

Response time measurements:

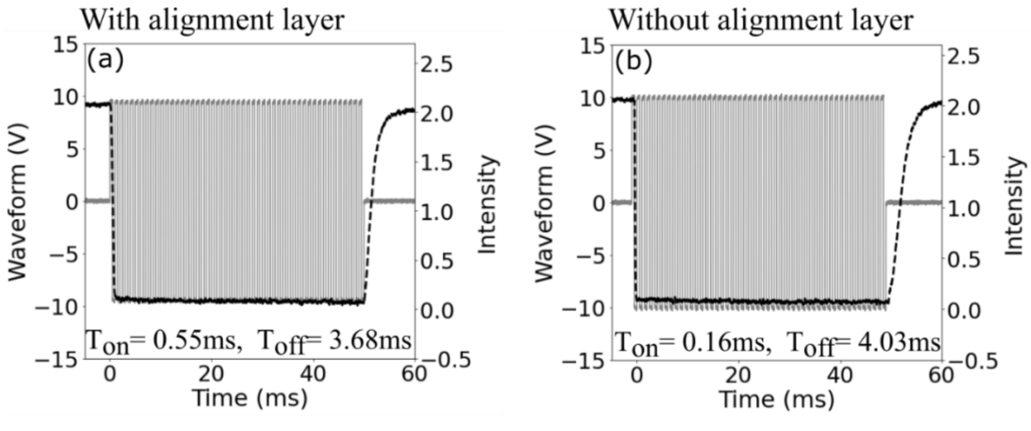

Fig. S10. Response time T_{on} and T_{off} for the nano-rectangle MS-LC cell: The switching performance of the sample (voltage dependent transmitted intensity- black curve) and the applied signals (gray curve) for a 750 nm cell (a) with top electrode alignment layer (T_{on}= 0.55 ms, T_{off} = 3.68 ms) and (b) without an alignment layer (T_{on}= 0.16 ms, T_{off} = 4.03 ms) for DC balanced driving. The voltage applied is 10 Vpp with a fixed frequency of 1 KHz ($\Delta\varepsilon$= 9.8 for the DFLC).
